# Expression of COX-2, NF-*κ*B-p65, NF-*κ*B-p50 and IKK*α* in malignant and adjacent normal human colorectal tissue

**DOI:** 10.1038/sj.bjc.6605120

**Published:** 2009-06-09

**Authors:** M P Charalambous, T Lightfoot, V Speirs, K Horgan, N J Gooderham

**Affiliations:** 1 Leeds Institute of Molecular Medicine, Wellcome Trust, Brenner Building, St James's University Hospital, Beckett Street, Leeds LS9 7TK, UK; 2 Department of Health Sciences, Alcuin College, University of York, York, UK; 3 Department of Surgery, Leeds General Infirmary, Great George Street, Leeds LS1 3EX, UK; 4 Biomolecular Medicine, Imperial College London, Sir Alexander Fleming Building, London SW7 2AZ, UK

**Keywords:** colorectal cancer, cyclooxygenase-2, fibroblasts, macrophages, nuclear factor-*κ*B, vascular endothelial cells

## Abstract

**BACKGROUND:**

Cyclooxygenase-2 (COX-2) is selectively over-expressed in colorectal tumours. The mechanism of COX-2 induction in these tumours is not fully understood, although evidence suggests a possible link between nuclear factor (NF)-*κ*B and COX-2. We hypothesised an association between COX-2 expression and NF-*κ*B-p65, NF-*κ*B-p50 and I*κ*B-kinase-*α* (IKK*α*) in both epithelial and stromal cells in human colorectal cancer.

**Methods:**

Using immunohistochemistry, we measured COX-2, NF-*κ*B-p65, NF-*κ*B-p65 nuclear localisation sequence (NLS), NF-*κ*B-p50, NF-*κ*B-p50 NLS and IKK*α* protein expression in matched colorectal biopsy samples comprising both non-tumour and adjacent tumour tissue from 32 patients with colorectal cancer.

**Results:**

We have shown that stromal cells of malignant and surrounding normal colorectal tissue express COX-2. In all cell types of malignant tissue, and in vascular endothelial cells (VECs) of neighbouring normal tissue, COX-2 expression was strongly associated with NF-*κ*B-p65 expression (Pearson's correlation, *P*=0.019 for macrophages, *P*=0.001 for VECs, *P*=0.002 for fibroblasts (malignant tissue), and *P*=0.011 for VECs (non-malignant tissue)) but not NF-*κ*B-p50 or IKK*α*.

**Conclusions:**

These data suggest that in these cells COX-2 induction may be mediated through activation of the canonical NF-*κ*B pathway. Finally, the lack of association between COX-2, NF-*κ*B-p65 or IKK*α* in stromal cells with the clinical severity of colorectal cancer as determined by Duke's stage, suggests that COX-2, NF-*κ*B-p65 and IKK*α* expression are possibly early post-initiation events, which could be involved in tumour progression.

Adenocarcinoma of the colon and rectum is the second leading cause of death from cancer in the industrialised world (Midgley and Kerr, 1999). The high prevalence of the disease is a driver for understanding the underlying molecular mechanisms of colorectal carcinogenesis.

Several epidemiological studies have reported a 40–50% decrease in the relative risk of colorectal cancer in persons chronically using non-steroidal anti-inflammatory drugs (NSAIDs) (Thun *et al*, 1991; Marnett, 1992; Giovannucci *et al*, 1995), indicating that these drugs may have a chemoprotective and possibly chemotherapeutic effect. Data from human studies, have shown that the NSAID, sulindac, can reduce the number and size of polyps in patients with familial adenomatous polyposis (FAP) (Waddell and Loughry, 1983; Giardiello *et al*, 1993).

Most NSAIDs in current use inhibit the action of cyclooxygenase (COX), a key enzyme in the production of prostaglandins (PGs). Cyclooxygenases are intracellular enzymes that catalyse the conversion of arachidonic acid to PGs and related eicosanoids. Isoforms of the COX-2 gene have been identified, which encode for the constitutively expressed COX-1 and the inducible COX-2. In the last decade, several studies have indicated a link between the expression of COX-2 and the pathogenesis of several types of human cancers, including breast (Brueggemeier *et al*, 1999; Half *et al*, 2002), gastric (Ristimäki *et al*, 1997), lung (Hida *et al*, 1998) and colorectal adenocarcinomas (Eberhart *et al* 1994; Tomozawa *et al*, 2000).

Although enhanced COX-2 expression in colorectal cancer tissues has been widely observed, the mechanisms that regulate the expression of COX-2 in colorectal tumours are not completely understood. Cyclooxygenase-2 expression can be induced by a variety of stumuli, including oncogenic viruses, growth factors, tumour promoters and cytokines.

Sequence analysis of the 5′-flanking region of the COX-2 gene shows two nuclear factor-*κ*B (NF-*κ*B) sites (Tazawa *et al*, 1994). *In vitro* inhibition of this protein has been shown to attenuate COX-2 expression in colorectal cancer cells, indicating that NF-*κ*B may play an important role in COX-2 induction (Plummer *et al*, 1999; Williams *et al*, 2003). Earlier, we have shown that upregulation of COX-2 is accompanied by increased expression of NF-*κ*B-p65 and I*κ*B-kinase-alpha (IKK*α*) in human colorectal cancer epithelial cells (Charalambous *et al*, 2003).

Recent studies have shown that tumour stroma also contributes to enhanced COX-2 expression in colorectal cancer. Increased levels of COX-2 have been localised in macrophages (Sheehan *et al*, 2003; Liu *et al*, 2005), fibroblasts (Sonoshita *et al*, 2002; Adegboyega *et al*, 2004) and vascular endothelial cells (VECs) (Brown and DuBois, 2005), indicating that both host and tumour cells may contribute to the production of PGs within the tumour microenvironment and the subsequent development of cancer growth.

We have therefore studied tissue biopsies obtained from patients with diagnosed primary colorectal carcinoma undergoing surgical treatment for their disease, for differences in expression of COX-2 in epithelial and stromal cells (macrophages, fibroblasts and VECs), in malignant and adjacent normal colorectal tissue, and for alterations in the expression of the upstream intracellular proteins, which seem to be associated to COX-2 expression, namely NF-*κ*B-p65, NF-*κ*B-p50 and IKK*α*.

## Materials and methods

### Patients

The study adhered to the tenets of the Declaration of Helsinki. Surgical specimens of primary tumours were obtained with informed consent from 32 patients (21 men and 11 women; aged 44–80 years, mean age 64.0 years±1.63 s.e.m.), with histologically verified colorectal cancer, treated at the Department of Surgery, York District Hospital, York, UK. Ethical approval for the study was obtained from the Human Research Ethics Committee at York District Hospital. A total of 17 patients had colon cancer and 15 had rectal cancer. Tumours were classified according to the Duke's classification (see Table 1). A total of 5 patients had Duke's A, 12 had Duke's B and 10 had Duke's C. The entire study was carried out blind using coded tissue sections.

### Tissue specimens

Tissue samples taken at operation for histopathological confirmation of disease were fixed in 4% buffered formaldehyde and embedded in paraffin wax; sections surplus to pathology requirements were made available for the study. For 23 patients, tissue sections of both normal and malignant colon or rectum were provided, whereas for nine patients only malignant tissue sections were available.

### Antibodies

The immunoglobulins (IgGs) used were as follows: (1) Goat polyclonal anti-human COX-2 (Santa Cruz Biotechnology; dilution 1 : 1500); (2) Rabbit polyclonal anti-human NF-*κ*B-p65 (Santa Cruz Biotechnology, Santa Cruz, CA, USA; dilution 1 : 1000); (3) Mouse monoclonal anti-human NF-*κ*B-p65 nuclear localisation sequence (NLS) (Chemicon Millipore, Watford, UK; dilution 1 : 100); (4) Mouse monoclonal anti-human IKK*α* (Santa Cruz Biotechnology; dilution 1 : 800); (5) Goat polyclonal anti-human NF-*κ*B-p50 (Santa Cruz biotechnology; dilution 1 : 200); and (6) Rabbit polyclonal anti-human NF-*κ*B-p50 NLS (Santa Cruz Biotechnology; dilution 1 : 100). All antisera were obtained from Santa Cruz Biotechnology.

### Immunohistochemistry

The expression of COX-2, NF-*κ*B-p65, NF-*κ*B-p65 NLS, NF-*κ*B-p50, NF-*κ*B-p50 NLS and IKK*α* in epithelial and stromal cells (macrophages, fibroblasts and VECs) of normal and malignant colorectal tissue was determined using a modified avidin–biotin immunohistochemistry procedure (Goggi *et al*, 1986). In preliminary experiments, each of the immunohistochemistry assays was optimised using a range of antisera dilutions (1/50–1/5000). For each assay, the negative control antisera (pre-immune sera) were confirmed negative for staining at the dilution optimised for the primary antibody and blocking peptides (Santa Cruz Biotechnology Inc., Santa Cruz, CA, USA) confirmed specificity. The sections were deparaffinised and rehydrated through xylene and a series of graded alcohol solutions. Endogenous peroxidase activity was blocked by immersing the sections into a solution of 3% hydrogen peroxide in distilled water for 30 min at room temperature, and then rinsed in cold running tap water for 10 min. Incubating the sections with 5% normal swine serum for 30 min at room temperature reduced non-specific background staining. Sections were then washed twice with phosphate buffered saline (PBS) and 1 ml of either the primary antibody or the normal goat or rabbit IgGs (negative control) was applied to each section, and left at 4°C overnight. The next day, the slides were washed twice with PBS, and then incubated with the secondary antibody solution (Biotinylated swine anti-goat, mouse, rabbit immunoglobulin; 1/150 dilution; 1 ml per section), for 1 h at room temperature. After being washed twice with PBS, they were incubated with the StrepABComplex solution (Dako Ltd, Ely, UK, 1 ml per section) for 1 h at room temperature, washed twice with PBS and immersed into the substrate (300 ml PBS, 90 *μ*l hydrogen peroxide and 2.5 ml 3,3-diaminobenzidine) for 3 min, and then rinsed with PBS and cold running tap water. Sections were then successively immersed into haematoxylin, acid alcohol and Scott's tap water to counterstain. Finally, the sections were dehydrated by successive immersion into 70% ethanol, 100% ethanol twice and xylene twice and mounted.

### Immunohistochemical evaluation

Processed specimens were scored under the light microscope and the intensity and extent of staining with COX-2, NF-*κ*B-p65, NF-*κ*B-p65 NLS, NF-*κ*B-p50, NF-*κ*B-p50 NLS and IKK*α* antibodies graded blind using coded slides. To assess and grade intensity and distribution of immunoreactivity in the colorectal stromal and epithelial cells, a scoring method, which has been described earlier was used (Yukawa *et al*, 1994). The distribution was scored according to the number of positive cells; none (not stained), 0; focal (<1/3 of cells stained), 1; multi-focal (1/3–2/3 of cells stained), 2; and diffuse (>2/3 stained), 3. The staining intensity was scored as: none (not stained), 0; mild (between 0 and 2), 1; and strong, 2. The distribution and intensity scored were added to produce the following grades for the staining: 0, negative; 2, weakly positive; and 3, 4 and 5, strongly positive. Sections treated with the normal goat or rabbit IgGs (negative controls) or omitting the primary antibody were devoid of staining. Positive staining controls for COX-2 included sections of kidney, uterus and brain.

### Statistical analysis

The Wilcoxon's signed rank test was used to compare the scoring of the respective immunoreactivity for COX-2, NF-*κ*B-p65 and IKK*α* between stromal cells of malignant and adjacent normal tissues. The Pearson's product-moment correlation coefficient test was used to assess the relation between COX-2 expression and NF-*κ*B-p65 and IKK*α*, and in addition to assess correlation between COX-2, NF-*κ*B-p65 and IKK*α* and the Duke's stages.

## Results

### Expression of COX-2 in normal and malignant colorectal tissue

**Figure 1:**
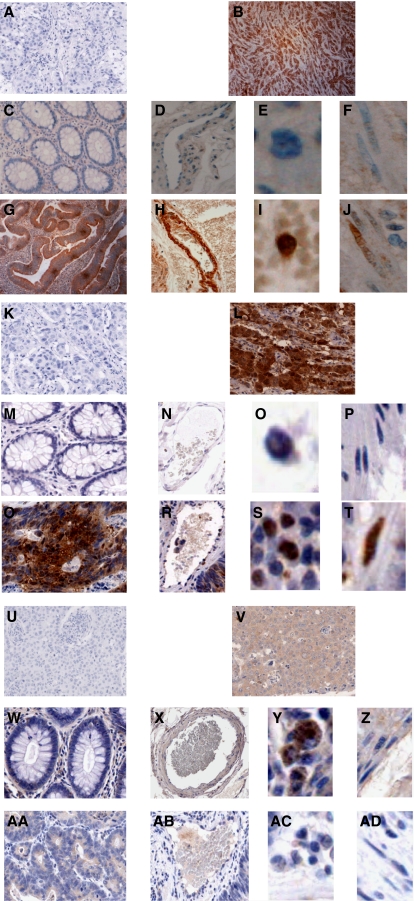
Immunohistochemical localisation of COX-2, NF-*κ*B-p65 and NF-*κ*B-p65 NLS in normal and malignant colorectal tissue from the same patient. The presence of the immunoreactive protein is indicated by brown staining. (**A**) Human malignant tissue treated with pre-immune sera as primary antibody (negative control for COX-2) (magnification × 10); (**B**) Human malignant tissue treated with anti-COX-2 antibody as primary antibody (positive control) (magnification × 10); (**C**) Epithelial cells, (**D**) VECs, (**E**) macrophages and (**F**) fibroblasts of normal colorectal tissue treated with anti-COX-2 primary antibody (magnification × 20 for epithelial cells, × 10 for VECs and × 40 for other two cell types); (**G**) Epithelial cells, (**H**) VECs, (**I**) macrophages and (**J**) fibroblasts of malignant colorectal tissue treated with anti-COX-2 primary antibody (magnification × 10 for epithelial cells, × 30 for VECs and × 50 for other two cell types); (**K**) Tissue treated with pre-immune sera as primary antibody (negative control for NF-*κ*B-p65) (magnification × 10); (**L**) Tissue treated with anti-NF-*κ*B-p65 antibody as primary antibody (positive control) (magnification × 10); (**M**) Epithelial cells, (**N**) VECs, (**O**) macrophages and (**P**) fibroblasts of normal colorectal tissue treated with anti-NF-*κ*B-p65 primary antibody (magnification × 20 for epithelial cells and VECs, × 40 for macrophages and × 30 for fibroblasts); (**Q**) Epithelial cells, (**R**) VECs, (**S**) macrophages and (**T**) fibroblasts of malignant colorectal tissue treated with anti-NF-*κ*B-p65 primary antibody (magnification × 10 for epithelial cells, × 20 for VECs and × 40 for other two cell types). (**U**) Tissue treated with pre-immune sera as primary antibody (negative control for NF-*κ*B-p65 NLS) (magnification × 10); (**V**) Tissue treated with anti-NF-*κ*B-p65 NLS antibody as primary antibody (positive control) (magnification × 20); (**W**) Epithelial cells, (**X**) VECs, (**Y**) macrophages and (**Z**) fibroblasts of normal colorectal tissue treated with anti-NF-*κ*B-p65 NLS primary antibody (magnification × 10 for epithelial cells and VECs, and × 40 for macrophages and fibroblasts); (**AA**) Epithelial cells, (**AB**) VECs, (**AC**) macrophages and (**AD**) fibroblasts of malignant colorectal tissue treated with anti-NF-*κ*B-p65 NLS primary antibody (magnification × 10 for epithelial cells, × 20 for VECs and × 40 for other two cell types).

**Figure 2:**
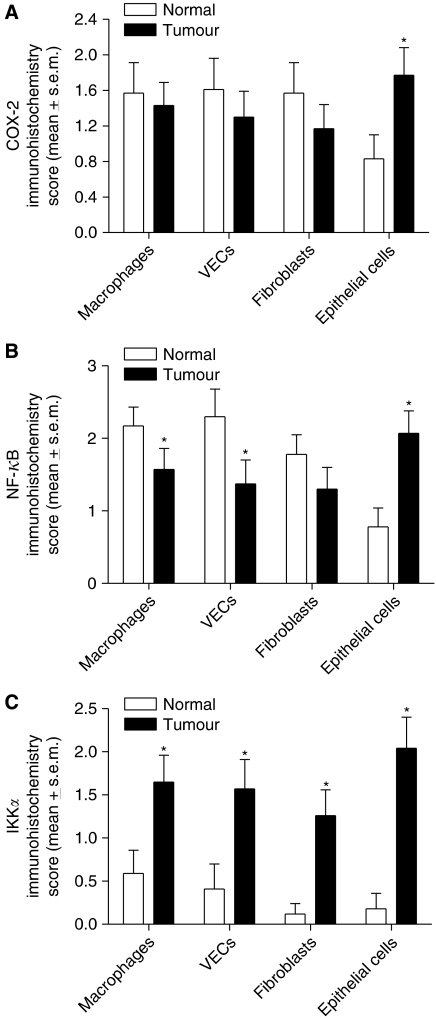
Expression of COX-2 (**A**), NF-*κ*B p65 (**B**) and IKK*α* (**C**) in matched normal and malignant colonic stromal and epithelial cells from 23 patients. ^*^Significantly different (Pearson's correlation test, *P*<0.03) from normal tissue.

Tissue sections of malignant and normal bowel from colorectal cancer patients were investigated for COX-2 expression by immunohistochemistry. Approximately, one-third of the patients strongly expressed immunoreactive COX-2 (score ⩾3) in stromal cells of both normal and malignant colorectal tissue (Figures 1 and 2A). Only 5 out of 23 patients strongly expressed immunoreactive COX-2 in non-neoplastic epithelial cells. In contrast, there was strong COX-2 expression in malignant epithelial cells in more than half of the patients (17 out of 30 patients). The expression was cytoplasmic. Statistical analysis of matched (normal *vs* malignant tissue from the same patient) samples showed no significant difference in the respective intensity scores of COX-2 of stromal cells in normal and malignant tissues (Wilcoxon's signed rank test; *n*=23; *P*=0.113 for fibroblasts, *P*=0.108 for macrophages, and *P*=0.066 for VECs). Cyclooxygenase-2 expression was significantly higher in malignant epithelial cells when compared with adjacent normal epithelium (Wilcoxon's signed rank test; *n*=23; *P*=0.003).

### Expression of NF-*κ*B-p65 in normal and malignant colorectal tissue

Tissue sections of normal and malignant bowel from colorectal patients were also investigated for NF-*κ*B-p65 expression. The majority of colorectal cancer patients strongly expressed immunoreactive NF-*κ*B-p65 (score ⩾3) in macrophages and VECs of normal colorectal tissue (Figures 1 and 2B). Moreover, about one-third of patients strongly expressed immunoreactive NF-*κ*B-p65 in normal fibroblasts, whereas only 3 out of 24 patients strongly expressed immunoreactive NF-*κ*B-p65 in normal epithelial cells. In cancerous tissue, more than half of the samples showed strong qNF-*κ*B-p65 expression in malignant epithelial cells, whereas only about a quarter to a third of the malignant tissues showed significant NF-*κ*B-p65 expression in stromal cells. In all cell types of both normal and malignant tissue, the NF-*κ*B-p65 staining was both cytoplasmic and nuclear. Cytoplasmic NF-*κ*B-p65 expression represented both inactive protein, which is bound to I*κ*B, as well as active NF-*κ*B-p65, which has been phosphorylated and released from I*κ*B, but which has still not translocated into the nucleus. Nuclear NF-*κ*B-p65 expression represented active protein. In order to determine whether any of the cytoplasmic NF-*κ*B-p65 protein was in its active form, we investigated the NF-*κ*B-p65 NLS expression in six patients who expressed significant cytoplasmic NF-*κ*B-p65 (Figure 1). The anti-NF-*κ*B-p65 NLS antibody specifically recognises an epitope overlapping the NLS of the p65 subunit of the NF-*κ*B heterodimer. This epitope is masked by I-*κ*B binding, and therefore the anti-NF-*κ*B-p65 NLS antibody selectively binds to I-*κ*B free, activated form of NF-*κ*B-p65 (Mitsiades *et al*, 2006). In all cases, a significant proportion of the cytoplasmic NF-*κ*B-p65 was found to be in its active form. In addition, we also confirmed that nuclear NF-*κ*B-p65 represented active protein. Statistical analysis of matched patient samples showed a significant reduction in the respective intensity scores of NF-*κ*B-p65 in macrophages and VECs of malignant tissues compared with those of normal adjacent colorectal tissue (Wilcoxon's signed rank test; *n*=24; *P*=0.027 for macrophages, and *P*=0.032 for VECs). No statistical difference was observed in similar analysis of fibroblasts (Wilcoxon's signed rank test; *n*=24; *P*=0.109). In contrast, NF-*κ*B-p65 expression was significantly higher in malignant epithelial cells when compared with adjacent normal epithelium (Wilcoxon's signed rank test; *n*=24; *P*=0.004).

### Expression of IKK*α*, and NF-*κ*B-p50 in normal and malignant colorectal tissue

**Figure 3:**
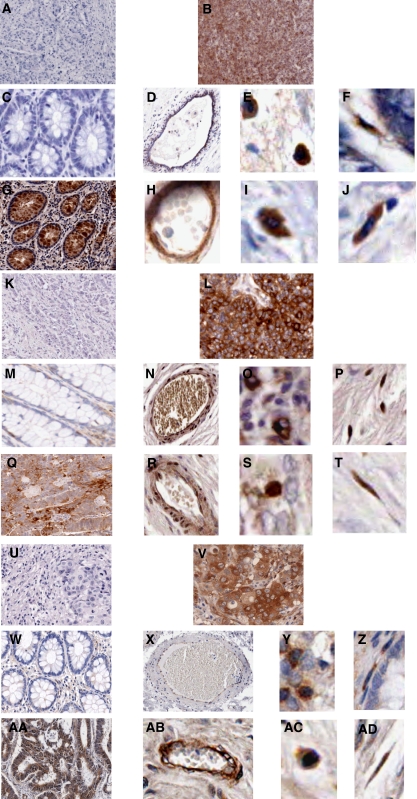
Immunohistochemical localisation of IKK*α*, NF-*κ*B-p50 and NF-*κ*B-p50 NLS in normal and malignant colorectal tissue from the same patient. The presence of the immunoreactive protein is indicated by brown staining. (**A**) Human malignant tissue treated with pre-immune sera as primary antibody (negative control for IKK*α*) (magnification × 10); (**B**) Human malignant tissue treated with anti-IKK*α* antibody as primary antibody (positive control) (magnification × 10); (**C**) Epithelial cells, (**D**) VECs, (**E**) macrophages and (**F**) fibroblasts of normal colorectal tissue treated with anti-IKK*α* primary antibody (magnification × 20 for epithelial cells, × 10 for VECs and × 80 for other two cell types); (**G**) Epithelial cells, (**H**) VECs, (**I**) macrophages and (**J**) fibroblasts of malignant colorectal tissue treated with anti-IKK*α* primary antibody (magnification × 10 for epithelial cells, × 20 for VECs and × 80 for other two cell types); (**K**) Tissue treated with pre-immune sera as primary antibody (negative control for NF-*κ*B-p50) (magnification × 10); (**L**) Tissue treated with anti-NF-*κ*B-p50 antibody as primary antibody (positive control) (magnification × 10); (**M**) Epithelial cells, (**N**) VECs, (**O**) macrophages and (**P**) fibroblasts of normal colorectal tissue treated with anti-NF-*κ*B-p50 primary antibody (magnification × 20 for epithelial cells and VECs, × 40 for other two cell types); (**Q**) Epithelial cells, (**R**) VECs, (**S**) macrophages and (**T**) fibroblasts of malignant colorectal tissue treated with anti-NF-*κ*B-p50 primary antibody (magnification × 10 for epithelial cells and VECs, × 40 for macrophages and × 50 for fibroblasts). (**U**) Tissue treated with pre-immune sera as primary antibody (negative control for NF-*κ*B-p50 NLS) (magnification × 10); (**V**) Tissue treated with anti-NF-*κ*B-p50 NLS antibody as primary antibody (positive control) (magnification × 10); (**W**) Epithelial cells, (**X**) VECs, (**Y**) macrophages and (**Z**) fibroblasts of normal colorectal tissue treated with anti-NF-*κ*B-p50 NLS primary antibody (magnification × 20 for epithelial cells, × 10 for VECs and × 40 for macrophages and fibroblasts); (**AA**) Epithelial cells, (**AB**) VECs, (**AC**) macrophages and (**AD**) fibroblasts of malignant colorectal tissue treated with anti-NF-*κ*B-p50 NLS primary antibody (magnification × 20 for epithelial cells and VECs, × 30 for macrophages and × 40 for fibroblasts).

There was little expression of immunoreactive IKK*α* in either stromal or epithelial cells of normal colorectal tissues, indicating that immunoreactive IKK*α* protein is not strongly expressed constitutively in these cells (Figure 2C and 3). However, there was a significant increase of IKK*α* expression in both stromal and epithelial cells of malignant colorectal tissues. The staining was purely cytoplasmic. Statistical analysis applied to matched patient samples showed a significant increase in the respective intensity scores of IKK*α* in both stromal and epithelial cells of malignant tissue, compared with those of normal colorectal tissue (Wilcoxon's signed rank test; *n*=18; *P*=0.010 for macrophages, *P*=0.006 for fibroblasts, *P*=0.028 for VECs, and *P*=0.005 for epithelial cells). Tissue sections of normal and malignant bowel from colorectal patients were also investigated for NF-*κ*B-p50 expression (Figure 3). A similar expression pattern to that of IKK*α* was observed for NF-*κ*B-p50 in both normal and malignant tissue. Most of the patients (7 out of 8) did not show any significant NF-*κ*B-p50 expression in either the stromal or epithelial cells of normal tissue. However, there was a significant increase of NF-*κ*B-p50 expression in both stromal and epithelial cells of malignant colorectal tissues. The staining was both cytoplasmic and nuclear. Cytoplasmic NF-*κ*B-p50 expression represented both inactive protein, which is bound to I*κ*B, as well as active NF-*κ*B-p50, which has been phosphorylated and released from I*κ*B, but which has still not translocated into the nucleus. Nuclear NF-*κ*B-p50 expression represented active protein. In order to determine whether any of the cytoplasmic NF-*κ*B-p50 protein was in its active form, we investigated the NF-*κ*B-p50 NLS expression in six patients who expressed significant cytoplasmic NF-*κ*B-p50 (Figure 3). In all cases, only a small proportion of the cytoplasmic NF-*κ*B-p50 was found to be in its active form. As there was available tissue from only eight patients to investigate the expression of this protein, no statistical analysis was performed.

### Co-expression of COX-2, NF-*κ*B-p65, NF-*κ*B-p50 and IKK*α* in normal colorectal tissue

**Figure 4:**
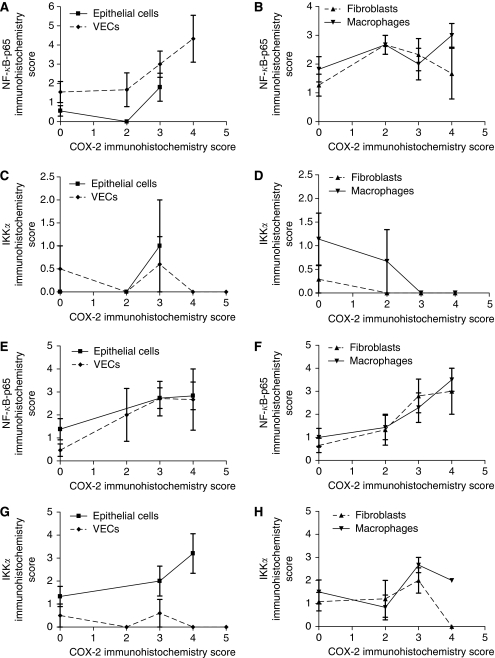
Expression of NF-*κ*B and IKK*α* compared with COX-2 in normal (**A**–**D**) and malignant colonic tissues (**E**–**H**). Values are mean±s.e.m.

In order to determine whether there was co-expression of COX-2 and NF-*κ*B-p65 or IKK*α* in normal colorectal tissue, serial sections were examined for expression of the four proteins. In the great majority of patients, strong (⩾3) COX-2 expression was accompanied by both cytoplasmic and nuclear NF-*κ*B-p65 expression in VECs (8 out of 9 patients) (Figure 4A). However, only a proportion of patients who expressed strong COX-2 levels in the other cell types within normal tissue, also expressed immunoreactive NF-*κ*B-p65 (4 out of 9 for fibroblasts; 5 out of 9 for macrophages; and 3 out of 5 for epithelial cells) (Figure 4A and B). In agreement with this histological finding, there was a significant correlation between COX-2 expression and NF-*κ*B-p65 expression in VECs (Pearson's correlation test, two-tailed, *P*=0.011, *n*=23), but not in the other cell types (*P*=0.192 for macrophages, *P*=0.171 for fibroblasts and *P*=0.111 for epithelial cells) (Figure 4A and B). Cyclooxygenase-2 expression in normal tissue did not correlate with either IKK*α* expression (Figure 4C and D) (1 out of 10 patients for macrophages; 1 out of 9 for VECs; 0 out of 11 for fibroblasts; and 1 out of 4 for epithelial cells) or NF-*κ*B-p50 expression (1 out of 5 patients for macrophages; 1 out of 4 for VECs; 0 out of 5 for fibroblasts; and 1 out of 3 for epithelial cells). In agreement with this histological finding, there was no correlation between the expression of COX-2 and IKK*α* in stromal cells of normal colorectal tissues (Pearson's correlation test, two-tailed, *n*=23, *P*=0.06 for macrophages, *P*=0.709 for VECs, and *P*=0.222 for fibroblasts) (Figure 4C and D).

### Co-expression of COX-2, NF-*κ*B-p65, NF-*κ*B-p50 and IKK*α* in malignant colorectal tissue

**Figure 5:**
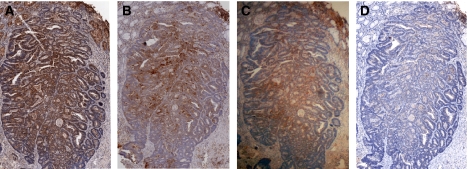
Serial sections of malignant tissue from the same patient showing co-expression of COX-2 (**A**), NF-*κ*B-p65 NLS (**B**), IKK*α* (**C**) and NF-*κ*B-p50 NLS (**D**) (magnification × 10).

Serial sections were also examined for co-expression of the four proteins in malignant colorectal tissue (Figure 5). In the majority of patients, COX-2 expression was accompanied by both cytoplasmic and nuclear NF-*κ*B-p65 expression in stromal cells (Figure 4E and F) (11 out of 16 patients for macrophages; and 9 out of 13 for VECs and fibroblasts). A total of 13 out of the 16 patients who expressed COX-2 in malignant epithelial cells, expressed NF-*κ*B-p65 (Figure 4E). In agreement with this histological finding, there was a significant correlation between COX-2 and NF-*κ*B-p65 expression in all cell types (Pearson's correlation test, two-tailed, *P*=0.019 for macrophages, *P*=0.001 for VECs, *P*=0.002 for fibroblasts and *P*=0.017 for epithelial cells) (Figure 4E and F). Only a proportion of patients who expressed significant COX-2 levels in stromal cells of malignant tissues, also expressed significant IKK*α* (Figure 4G and H) (3 out of 7 patients for macrophages; 4 out of 7 for VECs; and 2 out of 6 for fibroblasts) and NF-*κ*B-p50 levels (3 out of 5 patients for macrophages; 3 out of 5 for VECs; and 2 out of 5 for fibroblasts). Statistically, there was no correlation between COX-2 and IKK*α* in any of these cells in malignant tissues (Pearson's correlation test, two-tailed, *P*=0.322 for macrophages, *P*=0.378 for VECs, and *P*=0.578 for fibroblasts) (Figure 4G and H).

### Association between COX-2, NF-*κ*B-p65 or IKK*α* in stromal cells and severity of colorectal cancer

Comparison of the expression of COX-2, NF-*κ*B-p65 and IKK*α* in stromal cells of both normal and malignant epithelium and severity of colorectal cancer as determined by the Duke's stage, indicated that protein expression was not correlated with clinical assessment of disease severity (Pearson's correlation test, two-tailed; *n*=21 and *P*=0.13 for COX-2 in normal macrophages; *n*=21 and *P*=0.06 for COX-2 in normal VECs; *n*=21 and *P*=0.12 for COX-2 in normal fibroblasts; *n*=23 and *P*=0.15 for COX-2 in malignant macrophages; *n*=23 and *P*=0.20 for COX-2 in malignant VECs; *n*=23 and *P*=0.25 for COX-2 in malignant fibroblasts; *n*=21 and *P*=0.34 for NF-*κ*B-p65 in normal macrophages; *n*=21 and *P*=0.61 for NF-*κ*B-p65 in normal VECs; *n*=21 and *P*=0.47 for NF-*κ*B-p65 in normal fibroblasts; *n*=24 and *P*=0.41 for NF-*κ*B-p65 in malignant macrophages; *n*=24 and *P*=0.46 for NF-*κ*B-p65 in malignant VECs; *n*=24 and *P*=0.22 for NF-*κ*B-p65 in malignant fibroblasts; *n*=15 and *P*=0.07 for IKK*α* in normal macrophages; *n*=15 and *P*=0.95 for IKK*α* in normal VECs; *n*=15 and *P*=0.57 for IKK*α* in normal fibroblasts; *n*=19 and *P*=0.27 for IKK*α* in malignant macrophages; *n*=19 and *P*=0.51 for IKK*α* in malignant VECs; *n*=19 and *P*=0.91 for IKK*α* in malignant fibroblasts).

## Discussion

We found that both stromal and epithelial cells of malignant colorectal tissue express COX-2, indicating that both could contribute to the production of PGs within the tumour microenvironment. These results are in agreement with earlier studies, that found both stromal and epithelial colorectal cells expressing COX-2 in colorectal adenomas (Arnoletti *et al*, 2005; Pisano *et al*, 2005; Tatsu *et al*, 2005) and carcinomas (Battu *et al*, 1998; Yamashita *et al*, 2003; Ohta *et al*, 2006). We have reported earlier (Charalambous *et al*, 2003), a significant increase of COX-2 expression in malignant colorectal epithelial cells, compared with adjacent normal epithelium. We now report that this difference is not observed in stromal cells. In fact, COX-2 expression was higher in all three stromal cell types in normal colorectal tissue, compared with malignant tissue, although the difference in expression was not statistically significant. These results indicate that in malignant tissue COX-2 expression is predominantly epithelial, whereas in surrounding normal tissue it is predominantly stromal. This latter observation is in agreement with earlier reports suggesting that COX-2 expressed by stromal cells is directly involved in angiogenesis, preparing the surrounding colorectal tissue for local spread of malignant tumour (Williams *et al*, 2000; Sonoshita *et al*, 2002; Wendum *et al*, 2005).

Nuclear factor-*κ*B is an inducible eukaryotic transcription factor, which has a pivotal role in the regulation of the expression of numerous genes involved in immune and inflammatory responses (Sha, 1998; Bowie and O’Neil, 2000). In fact, NF-*κ*B is not a single protein, but a small family of closely related protein dimers, which bind to a common sequence motif known as the *κ*B site (Karin and Lin, 2002). Two regulatory pathways have been described that control the activity of these proteins: the canonical NF-*κ*B pathway, which is normally triggered in response to microbial and viral infections and exposure to pro-inflammatory cytokines; and the alternative pathway, which is triggered by certain members of the tumour necrosis factor (TNF) cytokine family (Karin and Lin, 2002). Nuclear factor-*κ*B-p65 is a member of the canonical pathway, whereas NF-*κ*B-p50 and IKK*α* are members of the alternative pathway. Once in the nucleus, NF-*κ*B can regulate several genes, including COX-2.

We have shown earlier that upregulation of COX-2 is accompanied by increased expression of NF-*κ*B-p65 and IKK*α* in malignant colorectal epithelial cells (Charalambous *et al*, 2003), supporting the proposal that NF-*κ*B is involved in COX-2 induction in these cells. This was in agreement with earlier model systems that showed expression of COX-2 was mediated by NF-*κ*B in human umbilical vein (Jones *et al*, 1993) and rheumatoid synoviocytes (Crofford *et al*, 1997). Subsequent *in vitro* studies provided further evidence in support of this hypothesis (Cherukuri *et al*, 2005; Duque *et al*, 2006; Konson *et al*, 2006). In this study, we have shown that NF-*κ*B-p65 and, to a lesser extent, NF-*κ*B-p50 are not only upregulated in malignant epithelial cells, but they are also significantly activated. Moreover, we have shown that in stromal cells (macrophages, fibroblasts and VECs) of malignant colorectal tissue, as well as in VECs of adjacent normal tissue, COX-2 expression is closely correlated with NF-*κ*B-p65, but not with IKK*α* or NF-*κ*B-p50. These results indicate that in these cells, COX-2 induction may be mediated primarily through activation of the canonical NF-*κ*B pathway in preference to the alternative pathway. These findings are in agreement with a recent study, which showed that COX-2 expression in colorectal cancer stromal cells, was associated with p-I*κ*B-*α*, another member of the NF-*κ*B canonical pathway (Vandoros *et al*, 2006). Interestingly, in normal tissue, COX-2 expression in macrophages and fibroblasts was apparently not associated with either NF-*κ*B-p65, IKK*α* or NF-*κ*B-p50.

In summary, we have shown that stromal cells of malignant and surrounding normal colorectal tissue express COX-2. In all cell types of malignant tissue, as well as in VECs of neighbouring normal tissue, COX-2 expression was strongly associated with NF-*κ*B-p65 expression but not IKK*α* or NF-*κ*B-p50, suggesting that in these cells, COX-2 induction may be mediated primarily through activation of the canonical NF-*κ*B pathway. Finally, the lack of association between COX-2, NF-*κ*B-p65 or IKK*α* in stromal cells with the clinical severity of colorectal cancer as determined by the Duke's stage, suggests that COX-2, NF-*κ*B-p65 and IKK*α* expression are possibly early post-initiation events, that could be involved in tumour progression.

## Figures and Tables

**Table 1:** Patient demographic information

**Age^a^**	**Sex**	**Tumour site**	**Duke's stage**	**Drug history**	**Tobacco use**	**Alcohol^b^**
72	M	Colon	B	None	No	8+
68	M	Colon	C	N/A	No	N/A
66	F	Colon	C1	5-Fluorouracil, Enalapril	No	0
69	M	Rectum	C	Co-codamol	No	1–7
49	F	Colon	B	N/A	Yes	N/A
63	F	Rectum	N/A	N/A	No	N/A
52	M	Rectum	C1	Adalat	No	1–7
75	M	Colon	C	N/A	No	N/A
68	M	Colon	B	Atenolol, Prednisolone, Warfarin, Diltiazem, Isosorbide, Gliclazide, Co-danthramer	No	1–7
69	M	Rectum	C1	Atenolol	No	8+
70	F	Rectum	B	Lithium, Thyroxine	No	8+
72	M	Rectum	B	Captopril, Naproxen, Allopurinol, Isosorbide, Frusemide, Atenolol, Prochlorperazine	No	1–7
78	M	Colon	A	N/A	No	N/A
56	M	Colon	A	None	No	8+
76	M	Colon	A	None	No	1–7
44	M	Colon	C	N/A	No	N/A
58	F	Colon	N/A	None	No	0
61	F	Rectum	A	N/A	No	N/A
66	F	Colon	B	None	No	0
54	M	Rectum	C1	None	No	1–7
49	M	Colon	B	None	No	8+
73	F	Colon	B	N/A	Yes	N/A
52	M	Rectum	B	None	No	8+
68	M	Colon	B	Salbutamol, Ferrous sulphate	No	0
63	F	Rectum	A	Salbutamol, Beclomethasone, Bendrofluazide	No	1–7
56	M	Colon	C	Losec	No	8+
68	M	Rectum	B	Sotalol, Aspirin	No	8+
80	F	Rectum	B	None	No	0
59	M	Rectum	C1	None	No	8+
66	M	Rectum	N/A	None	Yes	8+

N/A=not available.

a Age in years.

b Alcohol consumption in units per week (1 unit=half a pint of beer or one glass of wine or one shot of spirits).
